# The feasibility of the Prostate cancer: Evidence of Exercise and Nutrition Trial (PrEvENT) dietary and physical activity modifications: a qualitative study

**DOI:** 10.1186/s13063-017-1828-4

**Published:** 2017-03-07

**Authors:** Ellie Shingler, Lucy Hackshaw-McGeagh, Luke Robles, Raj Persad, Anthony Koupparis, Edward Rowe, Constance Shiridzinomwa, Amit Bahl, Richard M. Martin, J. Athene Lane

**Affiliations:** 1 University of Bristol, Bristol, UK; 2 Bristol Urological Institute, Bristol, UK; 3 North Bristol NHS Trust, Bristol, UK; 4 Bristol Haematology & Oncology Centre, Bristol, UK

**Keywords:** Feasibility, Framework analysis, Nutrition, Physical activity, Prostate cancer, Qualitative research

## Abstract

**Background:**

There is increasing evidence that low levels of physical activity and diets low in fruit and vegetables and high in meat and dairy products are risk factors for prostate cancer disease progression. The Prostate cancer: Evidence of Exercise and Nutrition Trial (PrEvENT) aimed to assess a diet and physical activity intervention in men undergoing radical prostatectomy for localized prostate cancer. The trial included a qualitative component to explore the experiences of men participating in the trial in order to understand the acceptability of the intervention and data collection methods. We report the qualitative findings of the trial and consider how these can be used to inform future research.

**Methods:**

PrEvENT involved randomizing men to either a dietary and/or physical activity intervention. Semi-structured interviews were conducted with a purposive sample of 17 men on completion of the 6 month trial. Interviews took place in clinic or as telephone interviews, if requested by the participant, and were audio recorded, transcribed, and analyzed using the thematic-based framework approach. Analysis was conducted throughout the data collection process to allow emergent themes to be further explored in subsequent interviews.

**Results:**

Three overarching themes were identified: acceptability of the intervention, acceptability of the data collection methods and trial logistics. Participants were predominantly positive about both the dietary and physical activity interventions and most men found the methods of data collection appropriate. Recommendations for future trials include consideration of alternative physical activity options, such as cycling or gym sessions, increased information on portion sizes, the potential importance of including wives or partners in the dietary change process and the possibility of using the pedometer or other wearable technology as part of the physical activity intervention.

**Conclusions:**

We provide insight into the opinions and experiences of the acceptability of the PrEvENT diet and physical activity intervention from the participants themselves. The interventions delivered were acceptable to this sample of participants, as were the data collection methods utilized. We also highlight some considerations for further behavioural change interventions in prostate cancer and other similar populations.

**Trial registration:**

ISRCTN, ISRCTN99048944. Registered on 17 November 2014.

**Electronic supplementary material:**

The online version of this article (doi:10.1186/s13063-017-1828-4) contains supplementary material, which is available to authorized users.

## Background

Owing to the increased early detection of latent and slow-growing tumours in prostate cancer, the number of men living with this disease has increased over recent years [1]. This has led to an interest in lifestyle modifications that can be used for the tertiary prevention of morbidity and mortality due to prostate cancer and the associated treatments [2].

There is a growing body of evidence suggesting the potentially protective effect of some nutrients and food items on prostate cancer, such as legumes and fruit and vegetables containing lycopene, owing to their antioxidant qualities [3, 4]. Other dietary aspects, such as increased meat and dairy consumption, are considered potential risk factors [5]. Similarly, physical activity interventions, such as brisk walking and endurance training, have been shown to be associated with a reduced risk of disease progression [6]. Physical activity may reduce the risk of prostate cancer progression through reduction of adiposity and inflammation, as well as through changes in sex and metabolic hormones [7]. Physical activity has also been found to improve quality of life in cancer survivors, including those with prostate cancer [8] but, within the UK, is currently only recommended by the National Institute for Health and Care Excellence guidelines for men experiencing fatigue as an adverse effect of prostate cancer hormone therapy treatment [9]. The World Cancer Research Fund estimates that 9% of advanced prostate cancer cases are preventable through modification of lifestyle behaviours: this would account for 940 cases each year in the UK [10].

The Prostate cancer Evidence of Exercise and Nutrition Trial (PrEvENT) is a feasibility study in men undergoing radical prostatectomy as treatment for localized prostate cancer. It explores the feasibility of conducting a cohort study and nested randomized controlled trial of diet and physical activity modification to investigate the effect on disease recurrence and progression.

For behavioural change interventions to be successful at improving diet and physical activity levels, the interventions must be deemed acceptable by the participants. Interventions not found to be acceptable will be less likely to have successful implementation or long-term beneficial outcomes [11]. The aim of this qualitative phase of the trial was to explore the attitudes and views of men about the acceptability of the diet and physical activity interventions, as well as the tolerability and ease of use of the data collection tools. The analysis therefore focuses on the feasibility of the trial and whether any changes should be considered for future behavioural change trials in prostate cancer survivors.

## Methods

### Overall trial design

**Fig. 1:**
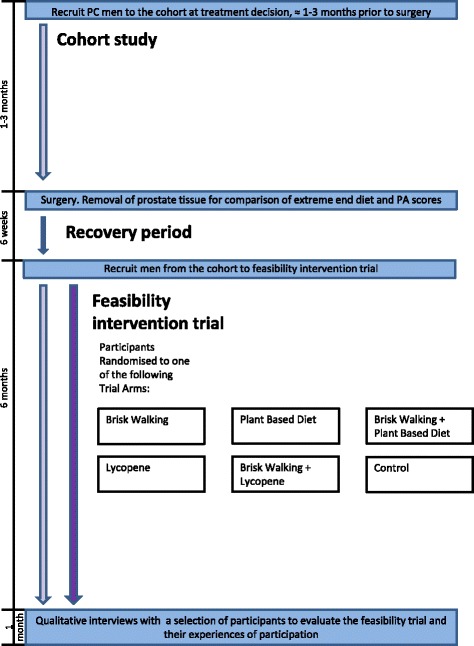
PrEvENT schematic

This qualitative study formed part of a randomized controlled trial, which assessed the feasibility and acceptability of diet and physical activity interventions for men after surgery for localized prostate cancer. The trial consisted of three phases, a cohort study, a nested randomized controlled trial and the qualitative study, a schematic of which can be seen in Fig. 1.

Participants were randomized to one of six arms in a factorial design, which comprised either a brisk walking intervention or physical activity control and one of two nutritional interventions (plant-based diet or lycopene supplementation) or nutrition control. Details of each of these components are detailed in Table 1.

**Table 1:** PrEvENT interventions

Intervention^a^	Allocation	Description
Physical activity	Brisk walking	Walking at a brisk pace for 30 min, at least 5 days a week, on top of normal physical activity
	Control	Carrying on with normal levels of physical activity
Nutrition	Lycopene supplement	Taking one lycopene capsule daily
	Plant-based diet	Eating as many portions of fruit and vegetables per day as possible, aiming for at least five daily portions. In addition, swapping dairy milk for non-dairy alternatives, for example soy milk, almond milk or rice milk, as often as possible
	Control	Carrying on with usual diet

^a^Interventions were delivered in a factorial design so that each participant was randomized to both a nutrition and a physical activity arm

Participants also received regular ‘motivational reminders’ from the trial team to encourage adherence. This contact was made via phone, email or post, depending on participant preferences, and provided motivational messages and resources such as recipes to encourage continued participation in the intervention. Full details of the PrEvENT interventions have been described in detail elsewhere [12]. The third phase of the trial involved semi-structured interviews with participants to explore their attitudes and views about the behavioural change modification and participation in the trial.

Trial data included self-report and objective measures of diet and physical activity. Self-report physical activity data were collected using the Recent Physical Activity Questionnaire [13] and self-report nutrition data were collected using a food frequency questionnaire [14] at baseline, and 3 and 6 months follow-up. Objective physical activity data were collected via two monitors, a pedometer worn daily throughout the 6 month intervention and an accelerometer worn for two periods of 1 week each at baseline and 6 months follow-up. Here, we report on the findings from the final phase of this trial, the qualitative interviews.

### Sample selection

Participants were invited to take part in an interview following the completion of all outcome measures at their final follow-up appointment, 6 months after randomization. Purposive sampling was employed to ensure maximum variation across the intervention arms and to ensure that the sample consisted of heterogeneous demographic characteristics, such as age, employment status, and educational level [15] to reflect the overall trial sample. Table 2 outlines the sample characteristics.

**Table 2:** Participant characteristics

Characteristic		*n* = 17	
		*n* or mean	% or standard deviation
Age (years)		66	5.49
Ethnicity	White British, white other	16	94
	Caribbean	1	6
Education level	Secondary school	9	53
	Further education	1	6
	University	7	41
Occupation status	Retired	12	71
	Employed	5	29
Trial arm	Lycopene, physical activity control	3	18
	Plant-based diet, physical activity control	3	18
	Brisk walking, diet control	3	18
	Lycopene and walking	4	23
	Diet and walking	3	18
	Control	1	6

### Data collection

Semi-structured interviews were primarily conducted face-to-face within the clinic (*n* = 12). For those who were unable to attend a face-to-face interview, a telephone interview was arranged (*n* = 5). All interviews were conducted between April 2015 and May 2016 and were audio recorded. Interviews were primarily conducted by ES (*n* = 9) and LHM (*n* = 7), with the exception of one interview conducted by LR. All interviewers were trained and experienced in conducting qualitative interviews and followed a pre-defined interview topic guide covering trial logistics, intervention specifics and overall experience of taking part in the trial. Examples of some of the questions set out in the topic guide can be seen in Additional file 1. Open discussion was encouraged and participants were prompted to elaborate on specific areas of interest. As interviews were conducted by researchers who were also implementing the trial, this enabled researchers to identify certain issues that could be included in the interviews and aided immersion in the data. Researchers aimed to minimize the potential bias that this approach can introduce through use of the interview topic guide and a focus on how the qualitative data can aid in improving future trials by exploring both negative and positive data that emerged.

### Data analysis

Interview audio recordings were transcribed verbatim for analysis by an external transcription company approved to process data subject to the Data Protection Act, anonymized and stored securely. Data were analyzed using the framework approach [15] assisted by NVivo 10 software [16]. This approach involved creating an initial coding index based on the interview topic guide and using this coding index to sort the data into themes. However, an inductive approach was used during analysis, allowing the coding and emergent themes to evolve throughout the analysis process. Interview transcripts were initially coded by one researcher (ES) and reviewed by a second researcher (LR) to ensure consistency. Any inconsistencies found were discussed and resolved between the two researchers. Emergent themes were reviewed and discussed regularly by both researchers to ensure that they remained grounded in the original data. Analysis was conducted in an ongoing manner throughout the data collection process to allow any emergent themes to be further explored in subsequent interviews. This also allowed researchers to identify when data saturation (i.e., no new themes or additional information emerging from the interviews) had been reached. The interviewing phase ceased once the researchers agreed that no additional information was emerging from the interviews as data saturation had been reached. A framework matrix was created that summarized each participants’ feedback on each theme and sub-theme.

## Results

A total of 25 participants were approached, of whom 17 (68%) agreed to participate. Reasons given for not consenting were: did not have time on the day of the clinic (*n* = 3), interviewer was not available on the day of the clinic (*n* = 2) and patient was too unwell (*n* = 1). No reason was given by two trial participants.

**Fig. 2:**
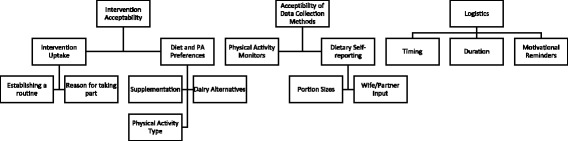
PrEvENT feasibility qualitative analysis – theme schematic. PA, physical activity

Three overarching themes were identified from the analysis: (1) intervention acceptability; (2) acceptability of the data collection methods and (3) trial logistics. A schematic of the themes and sub-themes identified is given in Fig. 2. The findings within each of these themes are discussed in detail next.

### Intervention acceptability

#### Uptake of interventions

In general, men suggested that the diet and physical activity interventions were easy to accommodate and did not infringe too much on their daily activity.

*I found it fairly easy… In the main, I find that I manage to eat sufficient – more often than not I manage to eat five portions of fruit and vegetables a day. – 13, Diet*

Additionally, it was suggested that the lifestyle change had become part of the daily routine or the new norm by eight of the participants; this can be seen across each of the active intervention arms.

*I think after you’ve established a routine with it, it becomes very easy. – 03, Lycopene*

*Well, that’s the important thing with these sorts of activity, is trying to build it into your routine. – 09, Brisk walking*

Men reported that one of the reasons they took part in the research initially was because the interventions were deemed ‘easy’ to do and that it was a simple way to contribute to prostate cancer research.

*Really, if it helps people understand what goes on… It didn’t require me to do very much. I could take part and I could take part usefully without having to do some massive change in lifestyle or whatever. It seemed worth doing. – 10, Brisk walking*

#### Diet and physical activity preferences

Although the participants stated a willingness to comply with all intervention arms prior to being randomized, at interview some expressed a preference over what kind of dietary and physical activity changes they would have liked to have made or would continue with following completion of the trial.

For example, men expressed mixed views over their preference to obtaining nutrients such as lycopene through supplementation. Some felt that taking a supplement was an easier option:

*I think I would probably struggle because I am not a great fruit eater… The (lycopene) tablets were fine. I get no trouble with tablets. – 08, Lycopene & walking*

*The lycopene was absolutely… You know, it’s taking a tablet. That’s absolutely fine… As far as taking any natural product, you know, providing it is a natural product, it’s there in nature anyway. – 05, Lycopene & walking*

In contrast, other participants viewed dietary intake as natural but not supplementation:

*I did say I would (take the supplement), but I would much prefer to have got it naturally.– 06, Brisk Walking*

Of the six men randomized to the plant-based diet, four suggested that they found dairy-free milk substitutes such as soy and almond milk acceptable alternatives to dairy milk. Additionally, three also stated that they found dairy-free milk to be less palatable when added to tea and coffee.

*I’d gone onto soya milk, but then, I just stopped it altogether… It just doesn’t make a nice cup of tea. – 02, Diet*

With regards to the physical activity arm, three of the ten men taking part in a brisk walking arm suggested that they preferred cycling to walking and that this was something they did alongside the walking intervention:

*I’m not a walker. I’m more of a cyclist, so it was a change of… well, I’d have to say, ‘Lifestyle’, but a change of leisure time, really. – 10, Brisk walking*

In addition, two men from the physical activity control arms advised that cycling was something they had done in the past.

### Acceptability of diet and physical activity measures

#### Dietary self-report measures

Men, in general, felt that they were able to complete the food frequency questionnaires successfully, although some voiced difficulties about giving representative answers about their food intake:

*Yes, a lot of it was about the food and that sort of thing, and, yes, just, sort of, trying to work out what to put was quite difficult. I mean, how many times do I eat beef in a month… and, you know, and with different vegetables? Yes, I don’t know how accurate it was, but I think I tried to be as objective and as accurate as I could. – 09, Brisk walking*

A number of participants found it difficult to work out exactly what classified as a portion size within the plant-based diet arms.

*It was also a bit confusing about the portion size of those vegetables and that sort of thing… I think, for blokes, it’s a bit, ‘A portion is a portion.’ You know, no matter. – 02, Diet*

Recurring references were made to having a wife or partner help confirm how many portions they had consumed, indicating the important role partners often play in dietary provision.

*I sat down with my wife actually and she could remember we had carrots twice a week or whatever. I needed a bit of help on that because you can't always remember. – 14, Lycopene & walking*

*Some of the portions that I put down, you know; my wife went through it after me and she said, ‘That’s rubbish you’ve put down there,’ and it was. – 16, Diet & walking*

In contrast to this, one participant who lived alone felt he was able to answer the questions accurately, owing to the control he had over his own food preparation.

*Because I live on my own, I do the shopping, obviously, and the cooking. So, I more or less know exactly what I’m eating or shopping. – 15, Lycopene*

#### Physical activity monitoring

Most felt that they were able to wear the physical activity monitors provided and record their daily steps. Two participants even stated that they found the monitors to be useful motivational tools and were purchasing their own at the end of the trial so that they could continue to monitor their daily steps:

*Respondent: And I can maybe buy myself a baby pedometer to play with, you know… I was shedding tears giving away my… (Laughter)*

*Interviewer: (Laughter) You bonded with it.*

*Respondent: Yes, that’s right. – 03, Lycopene*

Some also expressed an interest in monitors that could measure more than just steps and could provide further information, such as calories burned.

For some, the data monitoring provided the opportunity for self-reflection and also acted as a motivational tool.

*Even the log was not so bad because it was slightly reflective, so I could go, ‘Oh, what have I been doing today?’ – 07, Diet & walking*

*I got into the habit of doing it [the daily monitoring], yes, because that was part of the incentive to, sort of, or the drive to make me do it. – 09, Brisk walking*

However, ten participants also reported that the pedometers could, at times, be a ‘nuisance’ or ‘irritating’ to wear. The most common issue reported was that the pedometers came unclipped from trousers or belts as they were easily knocked off, which resulted in their loss or breakage.

*Because every now and then, you’re knocking against something, and it comes off. And a couple of times, I thought, ‘Oh God, I've lost it.’ It was in the car, or somewhere. – 04, Brisk walking*

### Trial logistics

#### Duration of intervention

Opinions were divided as to whether the men would have been able to continue with the trial for a further 6 months if the study had been extended to a year-long intervention. Some felt that, as they were in the routine of monitoring their daily activities, they would have been able to continue.

*It becomes so much a part of your routine that you just accept it, you know? – 03, Lycopene*

Others expressed concern that it would have been too long to continue for.

*I think it would become a bit of a chore, doing it for a year, just the recording. – 09, Brisk walking*

This distinction between being able to continue with the intervention but not the monitoring was made by other participants in other trial arms.

*I have to say, when I got to the end of the 6 months. I thought, ‘I haven't got to fill that form out every day and I haven’t got to record this and record that,’… I’m still carrying on doing it [changes to diet and walking] but of course not recording it is the… beauty as I see it. – 16, Diet & walking*

#### Timing of approaching patients

When asked whether starting the intervention 6 weeks after surgery had worked for them, most indicated that the timing had been acceptable. Some also suggested that they had already started trying to increase their physical activity at this point, indicating that the trial fitted in well with their own readiness to become more active.

*I had already started doing – you know, to do as much as I could anyway, because I’d been told by the surgeon to do that. – 04, Brisk walking*

*So I think, you know, 4 to 6 weeks [after surgery] is probably a good time to start. In fact, I did start to get active anyway. I think I went for a swim, just a short one, after about 4½ weeks. – 09, Brisk walking*

A few referred to needing to wait for the catheter to come out, at 2 weeks after surgery:

*I would say it would be only after you had the catheter thing out… because walking with that in is not fun. – 04, Brisk walking*

#### Motivational reminders

Participants expressed opposing views about the delivery of the ‘motivational reminders’ that formed part of the intervention. Some men felt that it was not required, although most admitted they could see why it was done:

*I think probably that, on balance, it’s probably a good thing to do. Did it help me? Probably not, because I was, sort of, fairly enthusiastic about keeping that going anyway. – 09, Brisk walking*

*So I mean, I think probably I didn't need the calls… So in that sense they were slightly irritating, but I completely understand why you would do it in general. – 04, Brisk walking*

For others, the additional contact, particularly with the research nurse was a positive experience:

*It just made me feel like you were appreciating my involvement really and they were keeping in touch all the time as to what was going on, sort of thing. You know, I wasn’t just away from the people who were doing the research, that I was involved in it.* – *15, Lycopene*

## Discussion

Participants were predominantly positive with regards to the acceptability of both the nutrition and physical activity interventions, with men indicating that they felt enabled to make the changes requested and to sustain them following completion of the trial. No difference was found in relation to acceptability between those randomized to a single intervention and those randomized to both a physical activity and dietary intervention. The data shows a common level of satisfaction among participants across all interventions, both in combination and on their own, indicating the acceptability of implementing multi-faceted lifestyle interventions in this group. These results, combined with the low dropout rate from the full trial (data not yet published), indicate a high acceptability of the interventions within this patient group.

Objective physical activity monitoring through the use of pedometers and accelerometers is recognized as the gold standard for physical activity monitoring in research trials but only a limited number of post-cancer diagnosis studies report using them [8, 17]. In prostate cancer specific research, a recent review of physical activity interventions for the prevention of prostate cancer progression identified nine trials that included a physical activity intervention. Of these nine studies, one used an objective activity monitoring tool in the form of a pedometer and did not report on the monitor’s acceptability [2]. Qualitative data from PrEvENT suggest that the data collection methods were acceptable and appropriate for use in this population. However, the common problems found among participants when using the pedometers, in particular, highlight the need to explore other tools, for example wrist-worn pedometers, as opposed to the clip-on versions used in the current trial.

The utilization of both the pedometer and the self-monitoring forms as a tool to increase physical activity by some men, indicates that there may be scope to use these tools as part of the intervention itself, rather than solely for data collection. This technique has been used in other intervention designs [18, 19], although not in this population group. There has also been an increase in the use of commercially available wearable technology in recent physical activity research and the has already been shown to be acceptable in use with older men living with chronic disease [20] and have potential further use in interventions aimed at older participants [21]. This could be exploited in further trials in this population by, for example, giving those in the intervention arms goals to reach for each day with their pedometers or other wearable technology devices allowing for self-monitoring of behavioural change [22].

The acceptability of the timing of the intervention was of particular interest with potential clinical implications. There is much discussion in the literature about the optimum time to introduce behavioural change in those receiving a cancer diagnosis, with uncertainty existing about how diagnosis and treatment affect the likelihood of people being able to initiate change [23]. With such side effects as urinary incontinence affecting men who have undergone prostatectomy, there is interest in whether this would affect men’s opinions about making lifestyle changes, such as a new physical activity regime. Previous qualitative research has also indicated that healthcare professionals have differing opinions and concerns over the optimal timing of lifestyle advice, owing to concerns of overwhelming those who have recently received a cancer diagnosis (data not yet published). PrEvENT data suggest that men are receptive to change following surgery for prostate cancer and felt they were able to embark on a physical activity or nutrition intervention 6 weeks after radical prostatectomy, making this an acceptable timing for future interventions.

The variability in responses to duration of intervention combined with the opinions that the data monitoring might have been difficult to continue with for a year’s period may indicate that an alternative type of follow-up is required should the trial be rolled out to a 12 month intervention. For example, daily monitoring could cease at 6 months with a further questionnaire and 1 week physical monitor activity data collection occurring at the 9 or 12 month follow-up time points. Alternatively, a less user-intensive method of self-monitoring could be explored through further feasibility work, such as providing the option to use electronic resources as opposed to the paper forms used in the current study.

We identified some areas where more support could be offered to participants in future behaviour change trials. For example, visual tools to help participants understand portion sizes could have reduced confusion over what counts as a single portion. References made to the need for input from wives or partners to confirm the men’s dietary intake highlight that partners can often act as the gatekeepers and providers of food in this group of men. This supports previous findings from qualitative research conducted with men with prostate cancer and their partners, which found that men considered their partners an integral part of implementing dietary change, with partners either driving the change or forming part of a joint decision-making process around diet [24, 25]. Although a diagnosis of prostate cancer might increase a man’s interest in his diet and the effect it can have on prognosis, wives and partners often retain their role as the providers of food, with the ultimate control over what is eaten [26]. This draws attention to the potential importance of including partners in the training delivered on dietary interventions and utilizing them further in dietary interventions of male cancer survivors.

Preferences made by some men to the type of physical activity they do, particularly to cycling, highlighted the importance of considering a choice of exercise regime or a more varied regime for future studies in this population. This is further consolidated when triangulated with the physical activity questionnaire data as, at baseline, 12.5% of participants reported having cycled for pleasure in the previous 4 weeks and 32% reported completing conditioning exercises on a bike or rowing machine (data not yet published).

Although men’s opinions varied on the usefulness of the motivational reminders used as part of the intervention, these allowed the interventions to remain embedded in the theory that informed their design. It has been shown that interventions based on behavioural change theory are more effective at improving diet quality for cancer prevention [27]. Some participants found these a useful aspect of the intervention and this may be one reason the trial has thus far had very low loss to follow-up rates.

A number of limitations were identified; primarily the inability to capture the potential effect of clinician or research nurse interest in nutrition and physical activity on recruitment into the trial. Recruitment to PrEvENT was completed by one dedicated research nurse, meaning it was not possible to interview participants who had been recruited and trained in the behavioural intervention by a range of nurses or clinicians [28]. Furthermore, it would have been a useful insight to collect qualitative data from those who declined to take part in either the cohort or randomized controlled trial phase of the study as, although the decline rate was low, it would have helped further understand the feasibility of recruiting to the trial. As those interviewed had agreed to take part in the trial, this indicates a level of willingness to engage in behaviour change, meaning that the results might not capture the opinions of prostate cancer patients who are not as willing to implement lifestyle behavioural change. However, although men who declined to take part were offered an interview to discuss their reasons for doing so, none consented to take part in the interview. Conducting interviews with those who did not wish to take part in the randomized controlled trial would have added additional depth of understanding to the barriers to recruitment. Although not qualitatively assessed, routine screening and recruitment data were collected as part of the trial, which provide data on the recruitment rates and reasons for refusal to take part (data not yet published).

## Conclusions

This research provides insight into the opinions and experiences of the acceptability of PrEvENT from the participants themselves. When taken into account with the low drop out and high adherence levels of the trial, it suggests high acceptability of the intervention in this population.

A number of factors have been identified for consideration in future behavioural change interventions in male cancer survivors. These include improved fruit and vegetable portion counselling, inclusion of wives or partners in dietary interventions, and more acceptable pedometers, as well as increased utilization of wearable physical activity monitors as part of the intervention and, finally, increasing the options for exercise type within physical activity interventions. Consideration of these recommendations should further improve the acceptability of nutrition and physical activity interventions in men receiving treatment for prostate, and other, cancer. As improving acceptability of physical activity and nutrition interventions is important for successful implementation of trials, these findings should be considered for the design and implementation of behavioural change trials in prostate, and other, cancer survivors.

## Additional file

Additional file 1: Sample topic guide questions. A selection of sample interview questions used to collect the qualitative data. (DOCX 11 kb)

## Abbreviation

PrEvENT: Prostate Cancer: Evidence of Exercise and Nutrition Trial
