# Supplementary material for: The feasibility of the Prostate cancer: Evidence of Exercise and Nutrition Trial (PrEvENT) dietary and physical activity modifications: a qualitative study
Source: Trials. 2017 Mar 7;18:106. doi: 10.1186/s13063-017-1828-4 (PMC5340012; doi:10.1186/s13063-017-1828-4)
Supplement: Additional file 1: — Sample topic guide questions. A selection of sample interview questions used to collect the qualitative data. (DOCX 11 kb) [file 13063_2017_1828_MOESM1_ESM.docx]

**Additional File 1 – Sample Topic Guide Questions**

**Intervention specifics**

What were the positives of…(intervention arm)

Were there any negative elements of…(intervention arm)

We asked you to make changes to your behaviour approximately 6 weeks after surgery; do you think this was too soon / not soon enough?

What do you think would be the ideal time to make changes to behaviour?

**Trial Logistics**

How did you find the regular contact / reminders by the research nurse and research team?

**Overall experience**

How would you sum up your overall experience of taking part in the trial?

How would you improve or change the trial to make it better for future participants?

What made you agree to take part in the first place?
